# Design and Study of a Smart Cup for Monitoring the Arm and Hand Activity of Stroke Patients

**DOI:** 10.1109/JTEHM.2018.2853553

**Published:** 2018-08-30

**Authors:** Maxence Bobin, Margarita Anastassova, Mehdi Boukallel, Mehdi Ammi

**Affiliations:** LIMSI-CNRS91400OrsayFrance; CEA-LIST91120PalaiseauFrance

**Keywords:** Stroke, monitoring, Internet of Things, home, rehabilitation

## Abstract

This paper presents a new platform to monitor the arm and hand activity of stroke patients during rehabilitation exercises in the hospital and at home during their daily living activities. The platform provides relevant data to the therapist in order to assess the patients physical state and adapt the rehabilitation program if necessary. The platform consists of a self-contained smart cup that can be used to perform exercises that are similar to everyday tasks such as drinking. The first smart cup prototype, the design of which was based on interviews regarding the needs of therapists, contains various sensors that collect information about its orientation, the liquid level, its position compared to a reference target and tremors. The prototype also includes audio and visual displays that provide feedback to patients about their movements. Two studies were carried out in conjunction with healthcare professionals and patients. The first study focused on collecting feedback from healthcare professionals to assess the functionalities of the cup and to improve the prototype. Based on this paper, we designed an improved prototype and created a visualization tool for therapists. Finally, we carried out a preliminary study involving nine patients who had experienced an ischemic or hemorrhagic stroke in the previous 24 months. This preliminary study focused on assessing the usability and acceptability of the cup to the patients. The results showed that the cup was very well accepted by eight of the nine patients in monitoring their activity within a rehabilitation center or at home. Moreover, these eight patients had almost no concerns about the design of the cup and its usability.

## Graphical abstract

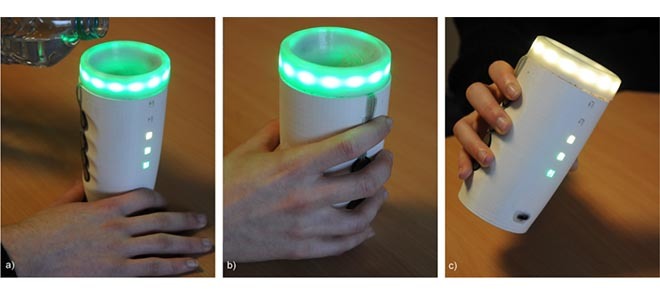

## I. Introduction

Strokes, also known as cerebrovascular accidents, affect 15 million people throughout the world each year [1], five million of whom die and five million of whom remain permanently disabled. After a stroke, motor deficits such as muscle weakness, spasticity and visual problems are often observed [2]–[4] and motor recovery is a time-consuming process, especially for the upper extremities and the hands. Stroke monitoring and rehabilitation are thus very expensive, since they require costly infrastructure and involve medical staff for long periods [5]. Moreover, demographic trends show that the number of stroke patients over 84 years old is expected to increase from 18% in 2005 to 48% in 2050 [6] and since the number of therapists will decrease proportionally, it will be not possible to treat all patients with the same efficiency [7]. Currently, patients attend hospital regularly to perform stroke-specific exercises under the supervision of a therapist, who assesses whether the exercise is performed accurately based on qualitative and subjective estimations (grasping an object correctly, shoulder position during grasping, etc.) [8]. Furthermore, when the patient has gained sufficient independence to return home, no monitoring is performed apart from subjective observations carried out during the remaining rehabilitation sessions.

The emergence over recent years of smart objects, wearables and, more generally, the Internet of Things allows the collection of qualitative and objective information about a patients motor functions for a low cost, and this can enhance the efficiency of the treatment of patients. These new platforms are based on common objects and contain several sensors and displays that can be used during a rehabilitation session to monitor the patients activity while performing exercises based on Activities of the Daily Living (ADLs) such as drinking, cooking, cleaning etc. [9]. In addition, these solutions provide consistent tools for the transparent monitoring of ADLs at home.

Although the implementation of a smart cup for health purposes has already been achieved, for example to diagnose pathogens such as the HSV2 virus [10], this paper presents an iterative process of design, implementation and validation for a smart cup designed to monitor stroke patients independence and recovery through ADLs, both in hospital and at home. The cup, called SyMPATHy, is a common object that can be used to simulate drinking exercises at the rehabilitation center or can be used by the patient to drink water, coffee and so on in daily life. SyMPATHy contains sensors that monitor stroke patients arm and hand activity and display feedback during usage. Firstly, SyMPATHy can provide relevant information on the way in which the patient fills, grasps, holds and manipulates the cup using liquid, pressure and movement sensors to determine the liquid level, the force applied on the cup, orientation or tremors during manipulation. SyMPATHy also contains embedded visual displays that can guide filling and manipulation. These displays provide feedback, helping patients to adjust their movements when visual or cognitive disabilities restrict perception of the environment. Secondly, the cup is able to detect the appearance and evolution of tremors. In fact, assessment of the evolution of tremors and their magnitude may lead to the detection of neurological or motor disorders [11]. A mobile application that can visualize all of this information (hand motor activity and tremor assessment) is also developed. This application provides improved knowledge about the patients recovery progress and thus independence in the everyday task of drinking, based on quantifiable information. Patient monitoring is not currently based on objective and qualitative information during rehabilitation sessions. Moreover, use of the cup at home, while the patient is alone, can provide useful feedback on the patients motor activity and independence, which are currently not monitored in any way. Therapists can thus assess any decline in the patients independence based on the daily basic task of drinking, and can then either adapt the arm and hand exercises performed during rehabilitation sessions or propose a readmission to hospital if necessary.

In this paper, we present a review of the existing platforms for rehabilitation of arm and hand motor function, monitoring and evaluation (Section II). The subsequent section (Section III) presents the design concept of the cup, including the data that are monitored and the sensory feedback provided. The first implementation of the prototype, inspired by interviews with health care professionals, is described, including the methods used for data collection and data processing and its electronic architecture (Section IV). Following this, an initial study involving health care professionals is presented that collects feedback on possible improvements to the SyMPATHy prototype (Section V). Based on the results of this study, a second prototype was developed, and the visualization application was presented to health care professionals with the aim of collecting feedback on these improvements (Section VI). Finally, a preliminary study was conducted with patients in order to obtain an evaluation of the usability and acceptability of the SyMPATHy cup (Section VII).

## II. Related Work

After a stroke, patients may face motor disorders such as hemiparesis, spasticity [12], visual impairments (vision loss, double vision, depth and distance perception problems or color detection problems) [4] or tremors [13], [14]. These motor and sensory disabilities have a direct impact on daily activities. Indeed, previous studies have shown that stroke patients experience problems in manipulating everyday objects (cups, forks, pens, etc.) [11], [15]. Patients tend to use compensatory strategies to reach a cup and move it to their mouth; for example, they may move their chest forward rather than extending their arm fully to reach and grasp an object. Gialanella *et al.* [9] have shown that ADLs are good outcome predictors in stroke patients, since they reflect the real motor activity of the patient by highlighting motor and sensory weaknesses. This section presents the state of the art in motor assessment tools for upper limbs after a stroke and new platforms for stroke monitoring.

### A. Motor Assessment Tools for Upper Limbs

Although various methods have emerged for evaluating the recovery of patients after a stroke, these are empirical and are based on visual estimations. Pandian and Arya [16] surveyed the existing motor assessment tools for evaluating upper extremity (UE) motor recovery. Most post-stroke hemiparetic patients recover their motor abilities in stages. After carrying out a series of longitudinal observations, Brunnstrom defined the two phases in arm and hand motor recovery called Brunnstrom Recovery Stages (BRS). BRS is divided in a part A for the arm and a part H for the hand [17]. BRS-A has seven stages that involve basic and complex arm controls, such as bending, extension or moving forward without moving the trunk. BRS-H has six stages that describe the recovery of grasping, and lateral and palmar prehension; the higher the stage, the better the recovery. However, BRS uses subjective observations made by the therapists. The Fugle-Meyer assessment (FMA) was created based on the BRS [18], and is the first stroke-specific assessment tool which follows the natural recovery process of a post-stroke hemiparetic patient [19]. Five domains are assessed: motor function, sensory function, balance, joint range of motion and joint pain. Each domain is divided into two parts, the UE and lower extremity (LE). However, the UE aspect of FMA (FMA-UE) is more widely used than FMA-LE [20]. Like the BRS, FMA is based on subjective observations. Following this, the Wolf motor function test (WMFT) was exclusively developed for patients receiving constraint-induced movement therapy. The WMFT is used to estimate a patients UE motor deficits, and involves movements from shoulder flexion to fine finger flexion [21]. However, the WMFT requires specific equipment, such as a dynamometer, to measure the prehension force, and can therefore be expensive. Finally, the most interesting scale is the action research arm test (ARAT), which is divided into four categories: grasp, grip, pinch and gross movements [22]. The ARAT includes 19 items that are given scores of between zero (cannot perform any part of the test) and three (performs the test normally). The patient is asked to complete the most difficult task in the subscale; if the patient achieves the maximum score in this task (score = 3), this score is assigned for this sub-scale and the patient then tries the next sub-scale. It should be noted that the ARAT grip and gross movements are more widely used than the grasp and pinch movements [23]. The interesting aspect of ARAT is that the test must be performed under standard conditions (with a specific chair and table set), meaning that the test is easily reproducible. Moreover, the objects used during the test can be easily implemented using sensors (a cube or cup, for example). All of the above estimation methods evaluate arm and hand function using different tasks and scales, although the most common tasks are hand grasp and elbow flexion. In addition, these empirical methods are based on visual estimations and subjective observations, which require the presence of a therapist.

### B. New Platforms for Stroke Monitoring

In the past decade, several authors have investigated new information and communication technologies for health applications, and more particularly to provide new approaches to the monitoring of stroke patients during rehabilitation. Iosa et al. found that smart objects and wearable devices will be able to enhance the monitoring of stroke patients in the near future [24]. For example, Patel *et al.* [25] used accelerometers to accurately estimate the scores assigned by clinicians using the functional ability scale. Other studies have tried to characterize tremors during daily activities using wearables, by comparing the number of movement units (NMUs); these are based on an analysis of the smoothness and efficiency of the movements made by healthy, mild stroke and moderate subjects [11], [26]. The results show that the more a patient is affected by stroke, the greater the number of NMUs that can be found in the movement. Some studies of wearables focus on monitoring the body kinematics of stroke patients in everyday life. Tognetti et al. proposed an innovative garment that is able to detect the position and movement of the upper limbs [27] while Laudanski *et al.* [28] used inertial sensors to estimate lower limb joint kinematics during stair ambulation in healthy older adults and stroke survivors. However, the patients were required to wear devices or sensors at specific locations on their body. Other works have investigated self-monitoring for rehabilitation and have found that this is effective in improving physical function and quality of life, particularly for early post-stroke patients [29]. For example, MagicMirror is a self-monitoring platform that based on motion tracking using a Kinect [7]. Rehabilitation exercises are performed with a therapist and recorded with a camera, and the patient then uses this video recording as a reference exercise while exercising at home. However, these exercises are stroke-specific and are not based on ADLs. Moreover, they also require the therapist to spend an entire session at the hospital recording the reference exercise.

As described above, current evaluation methods for motor activity recovery after a stroke are empirical and are based on visual estimations made by the therapists during rehabilitation sessions. Other solutions have emerged for monitoring stroke patients during everyday activities in order to assess their independence and recovery. However, wearables require the patient to wear sensors, and self-monitoring can be expensive and is based on specific exercises that do not match those performed in daily life. Using objects with embedded sensors to perform exercises based on ADLs is an interesting alternative to provide relevant information to a therapist about a patients level of independence in everyday life. The manipulation of a cup (filling, drinking, etc.) is a task that can be used to provide consistent information on hand and arm motor functions, as it involves reaching, grasping, filling and manipulation.

## III. Design Concept

A smart cup called SyMPATHy has been developed in order to provide information on a patients arm and hand motor activities during ADLs (drinking coffee in the morning, drinking water during lunch, etc.). SyMPATHy is based on two sub-tasks of ARAT: grasping a cup and transferring water from one cup to another. The information collected by SyMPATHy allows a therapist to assess a patients level of independence based on the daily task of drinking, both during therapy sessions and at home. In addition, SyMPATHy provides real-time sensory feedback to the patient.

The design process of SyMPATHy included five main steps: (i) identification of the task to be performed (Section III-A); determination of the information to monitor (Section III-B) and sensory feedback to provide to the patient (Section III-C); (ii) implementation of the prototype (Section IV); (iii) a preliminary study of the functionalities of SyMPATHy (Section V); (iv) improvements to the prototype and an insight into the visualization application (Section VI); and (v) a preliminary study involving patients on the usability and acceptability of the cup (Section VII).

### A. Task Identification

Timmermans *et al.* [30] showed that positioning and manipulating an object are the most suitable training tasks for stroke patients. These tasks require good coordination of the movements of the upper limbs, and are generally based on an action-perception loop exploiting several sensory channels (vision, tactile sensation, proprioception, audio). A typical activity involving positioning and manipulation is drinking; the patient needs to grasp the bottle and the cup, raise the bottle above the cup and control the amount of liquid poured into the cup. This activity involves motor actions of different parts of the upper limbs (hand, arm etc.). Moreover, it simultaneously involves visual, tactile, proprioception and audio sensory feedback. Several researchers have investigated the use of such tasks (manipulation of a cup, drinking from a cup) in assessment of a stroke patients progress [11], [15], [31], and have shown that kinematic measurements such as the total movement time, peak angular velocity of the elbow and measurements of compensatory trunk and arm movements can be used as an objective assessment of upper extremity motor recovery.

Based on this previous research, the current work focuses on the task of reaching, filling and transporting a cup, which uses various motor sub-tasks of the upper limb (arm movements, grasping, etc.). This task was identified from the literature and judged by two qualified health professionals working at a stroke rehabilitation center as being suitable to assess a patients independence. These health professionals were a medical doctor and an occupational therapist; they reported that using ADLs (manipulation of a cup for drinking) to design the platform would allow easier acceptance of the monitoring system.

### B. Monitored Information

Based on this ADL (cup filling and manipulation), we identified the main elements necessary to assess the patients recovery progress. These can be divided into two categories: (i) arm and hand motor activity, which includes the liquid level inside the cup, the grasping force applied on the cup, the orientation of the cup during manipulation and the placement of the cup on a specific target; and (ii) the appearance and evolution of tremors.

#### 1) Arm and Hand Motor Activity

The data collected for assessment of the patients arm and hand motor activity are presented following the action sequence when using a cup. Firstly, monitoring of the liquid level allows analysis of the quantity of liquid poured into the cup. Therapists can use this information to evaluate the accuracy and coordination of movements during filling, since pouring water into a cup poses a challenge for motor-deficient stroke patients.

Secondly, the pressure applied by the patient on the cup is monitored to evaluate the motor ability of the hand. Sunderland *et al.* [32] showed that the force applied during the grasping phase is an indicator of the recovery state of the hand function. Finger spasticity or finger motor disorder can be easily detected, and this can be merged with other information to assess motor disorders.

Thirdly, tracking the orientation of the cup during manipulation allows the therapist to understand the way in which the patient holds the cup (its verticality) and to evaluate the evolution of motor problems. Motor, sensory or cognitive disabilities often lead to incorrect posture in which the cup is not held vertically.

Finally, the movements of the cup are monitored in order to evaluate the overall coordination of the motor activity of the upper limbs. We focus particularly on the placement of the cup onto a specific target, for example a drinks coaster on the table. Monitoring the placement of the cup in a specific position allows assessment of 3D vision and coordination of movements (hand displacement, finger grasping, etc.) and detection of whether the patient can reach a specific area on the table.

#### 2) The Appearance and Evolution of Tremors

SyMPATHy can also detect tremors in a patients hand. Tremors are involuntary muscle contractions and relaxations involving oscillations or twitching movements of one or more body parts with low amplitude. Previous work has indicated that post-stroke tremors have a frequency of less than 5 Hz and are perpendicular to the direction of the movement [33]. Several researchers have also shown that changes in the frequency and magnitude of tremors allow detection of the evolution of neurological or motor disorders [11].

### C. Sensory Feedback

In order to determine the best feedback on the orientation, liquid level and the relative position of the cup, we held discussions with health care professionals (a medical doctor and an occupational therapist). The feedback used for cup orientation is visual feedback regarding the top of the cup, which indicates the angle of tilt, since audio feedback cannot easily provide information on the angle. A circle of fourteen LEDs is embedded into the top of the cup, underneath a semitransparent material, and these display colors according to the inclination of the cup. A set of discriminable colors based on standard usage in European countries (green, yellow, orange and red) are used to identify the orientation of the cup. Using a set of different colors helps to avoid ambiguity in the patients perception. The following color/tilt associations were used: green when the cup is held correctly (0 to 20°); yellow or orange for intermediate configurations (yellow: 20 to 35°, orange: 35 to 50°); and red if the cup is very tilted (> 50°).

Regarding the liquid level, the above discussion led to the use of a visual channel that displays colors vertically on the cup. These colors simplify the information display by providing a discrete representation of the liquid level. From a technical point of view, a vertical column of five LEDs is lit up from red to green, including orange and yellow, from the bottom to the top of the cup. The color choice is also based on standard usage in European countries.

Regarding the cup placement, binary audio feedback was used to indicate to the patient that the target has been reached. This allows avoiding overload the visual channel with another visual feedback. From a technical point of view, a speaker is integrated to the cup, and this plays a tone when the patient places the cup in the correct position.

## IV. Implementation of the Prototype

This section presents the different aspects of the implementation of the SyMPATHy prototype: (i) data collection; (ii) data processing; and (iii) hardware implementation.

### A. Data Collection and Sensors

SyMPATHy contains a series of embedded sensors that collect the data required to monitor the information described above. All collected data are sent to a remote computer via wireless communication. The functionalities presented below appear in the sequence of actions involved in a drinking task: filling, grasping, manipulating and releasing the cup.

#### 1) Liquid Level Detection

**FIGURE 1.**
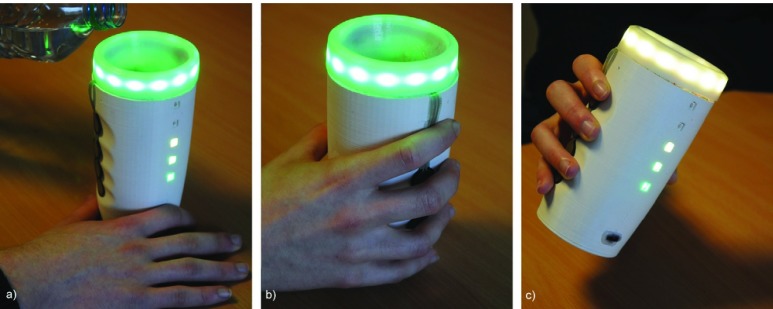
The SyMPATHy cup: (a) grasped on the handprint; (b) being filled with water; and (c) tilted during manipulation.

Due to the limitations of industrial liquid level sensors (low reactivity, size, etc.), SyMPATHy contains its own custom sensor based on measurements of the liquids conductivity. Five conductive electrodes are placed vertically inside the cup, corresponding to a volume of 100 mL. (Figure 1.a). Each electrode is connected to a 100K$\Omega $ resistance (chosen based on empirical measurements of different liquids) that creates a voltage divider bridge. An electrode wired to the ground is placed on the bottom of the cup. The electrodes act as switches, and the liquid closes the circuit. When liquid is poured into the cup, one or more voltage divider bridges are activated, modifying the resistances measured by the central unit. The liquid level sensor is divided into only four tension divider bridges, since higher precision is unnecessary.

#### 2) Detection of Grasping Force

SyMPATHy contains five embedded pressure sensors placed on the surface of the cup in the shape of a handprint (Figure 1.b). “Force Sensing Resistors” (FSR 402) have been used and provide the mean force (pressure) applied on the sensor’s surface.

#### 3) Orientation and Tremor Detection

SyMPATHy contains an embedded inertial measurement unit (IMU) that retrieves raw movement data. The nine-axis motion-tracking device used (Invensense MPU-9150) contains a three-axis accelerometer, a three-axis gyroscope and three-axis magnetometer. Data are sampled at 30 Hz so that LEDs are lit smoothly (Figure 1.c).

Data from the IMU sensor are used to calculate the orientation of the cup. Tremors are computed from the accelerometer and gyroscope in order to provide translational and rotational tremor information (Section IV-B2).

#### 4) Detection of the Cups Position

To detect whether the patient has successfully reached the target on the table, SyMPATHy contains an embedded Near Field Communication (NFC) reader (PN532 V3) and uses a NFC tag (MiFare Classic) as a target. NFC is a bi-directional technology that allows communication over a short range with mobile phones or computers, and does not require authentication. The shape of the antenna is a rectangle of size $40.4 \times 42.7$ mm, and the minimum vertical detection distance is around 5 mm due to the presence of material at the bottom of the cup. One or more NFC tags can be placed on the table to define different targets for the patient to reach.

### B. Data Processing

#### 1) Orientation Calculation

The orientation of the cup is calculated from the values returned by the IMU sensor. We used the RTIMULib-Arduino^1^ framework from RichardsTech to retrieve IMU data. This library also allows us to calibrate the accelerometer and the magnetometer and to compensate for gyroscope drift. When taking each reading, the gyroscopes bias is calculated and integrated into the calculation of the values. Based on the values returned by the IMU, the RTQF fusion algorithm is used to compute the position of the cup. This algorithm is a simplified version of a Kalman filter, which avoids matrix inversions and other intensive calculations for the effective fusion of data. RTQF linearly extrapolates between the previous and current orientations of the IMU using the gyroscope measurements and the time between samples. The accelerometer and magnetometer provide an absolute reference (pitch/roll and yaw, respectively) that enhances the accuracy of the prediction. At each step, the RTQF algorithm computes two quaternions: a predicted quaternion from the gyroscope measurements and a ground-frame-referenced measured quaternion from the accelerometer and magnetometer. Although the predicted quaternion is stable, it is subject to drift, whereas the measured quaternion is less stable but has no drift. The SLERP (spherical linear interpolation) technique is used to find an intermediate quaternion between the predicted and measured ones. The SLERP power is a coefficient of between zero and one, and controls towards which of the predicted or the measured quaternion, the resulting quaternion is. After empirical testing, we decided to set the SLERP power to 0.02, since this gives the best fusion results. If the gyroscope and the accelerometer measurements exceed their full-scale range of motion, the algorithm will take a few seconds to close the gap between the predicted state and the measured state. A low-pass filter is also applied to the signals to suppress noise. We set the cutoff frequencies of the low-pass filter to 20 Hz for the gyroscope and 21 Hz for the accelerometer, based on empirical measurements. Data signals over 20 Hz are unnecessary since stroke tremors have a frequency lower than 5 Hz [33]. Based on the quaternion computed by the RTQF algorithm, which corresponds to the 3D orientation of the cup, we calculated the tilt angle of the cup, corresponding to the angle between the cup and the vertical; in other words, this is the $\Phi $ angle expressed in a spherical coordinate frame. The color of the LEDs is then generated in order to provide the correct visual feedback to the patient.^1^https://github.com/richards-tech/RTIMULib-Arduino

#### 2) Tremor Analysis

Tremor analysis is based on spectral analysis tools provided by the FFTW library^2^: Fast Fourier Transform (FFT) and Power Spectral Density (PSD). We used the FFT algorithm, which computes the discrete Fourier transform (DFT) faster than the naive approach, to identify the frequency components of a noisy time domain signal. The naive DFT algorithm requires $\mathrm {O}(n^{2})$ operations, while the FFT algorithm requires only $\mathrm {O}(n \log (n))$. Firstly, for each axis of the accelerometer and the gyroscope, we compute the FFT $X$ of length N of a sequence $x_{n}$ based Cooley-Tukey algorithm:

\begin{equation*} X_{k} = \sum ^{N-1}_{n=0}{x_{n}e^{-\tfrac {2 \pi i}{N}nk}},\quad k = 0,\ldots ,N-1\tag{1}\end{equation*}

^2^http://fftw.org/

We then compute the PSD for each axis of the accelerometer and the gyroscope, which is a measurement of the energy at various frequencies that describes how the power of the signal is distributed over the frequency. The average power $P$ of a signal $x(t)$ over a period of time $T$ is given by the following time average:

\begin{equation*} P = \lim \limits _{T \rightarrow +\infty } \frac {1}{2T}\int _{-T}^{T}|x(t)|^{2}dt\tag{2}\end{equation*}

Subsequently, we seek the maximum value of the PSD, which corresponds to the fundamental frequency of the signal. Interpretation of the oscillations as post-stroke tremors is left to the therapist, based on the computed frequency and magnitude data.

### C. Hardware Implementation

The architecture of SyMPATHy is based on the Raspberry Pi Zero (RPi-Z). The RPi-Z is a tiny computer (65.0 mm $\times \,\, 31.0$ mm $\times 5$ mm) which contains a 1 GHz single-core CPU and 512 MB RAM. The RPi-Z is inexpensive ($5) and well suited for prototyping. Data from the IMU sensor are retrieved using I^2^C (inter-integrated circuit) which is a multi-master, multi-slave, single-ended serial computer bus. The serial peripheral interface (SPI) bus, which is a synchronous serial communication interface for short distance communication, is used to communicate with the NFC reader. Moreover, due to a lack of analog-to-digital converters (ADC) on the RPi-Z that can convert a continuous physical quantity (usually voltage) to a digital value, we added these to the RPi-Z to connect the liquid sensors and the force sensors. The NeoPixel RGB LEDs used for the visual display require a 5 V power supply and 60 mA per LED; SyMPATHy has 19 LEDs (five for the liquid level and 14 for orientation), requiring 1140 mA in total. Since the RPi-Z cannot deliver more than 5 V and 1 A, two PowerBoosts were added between the RPi-Z and each strip of LEDs. PowerBoosts supply an input voltage of between 1.8 and 5 V, and provide a constant 5 V output with a minimum output current of 1 A. SyMPATHy contains a 3.7 V 2500 mAh battery. A LIPO charger is also connected to the battery and all electronic components in order to allow battery recharging via a micro USB cable. Finally, a power switch allows the patient or therapist to power off the device.

Real-time wireless communication (Bluetooth and Wi-Fi) with a computer was considered for logging and processing data; however, this solution involves several drawbacks in terms of the usability and reliability of communication. For example, Bluetooth communication requires a short distance between the device and the hotspot, thus limiting the operational space. In addition, Bluetooth communication can cause a loss of data, making the cup less reliable for patient monitoring. Although Wi-Fi offers an extended range and a better data transfer rate, its main constraint in terms of real-time communication is its high power consumption, which poses an important challenge in the field of autonomous smart objects. To solve this issue, the first prototype of SyMPATHy logs data locally using separate files. A Wi-Fi dongle was added to the cup in order to enable communication with a remote computer. Since power consumption is the main issue, the saved data are transferred to the computer over Wi-Fi when SyMPATHy is plugged into a micro-USB connector. This configuration avoids wasteful power consumption and ensures more reliable data transfer. SyMPATHy can operate in this way for one day using LED indicators, and up to two days without visual feedback.

## V. Iteration 1: Preliminary Study of the Functionalities of SyMPATHy

### A. Aim of the Study

This study aimed to garner feedback about possible improvements to the functionalities of the cup, before testing it with post-stroke patients. The study also investigates the cup in terms of its usefulness, integration and acceptability to therapists. For this study, we made contact with health care professionals who worked in functional rehabilitation centers and medical centers. As these health care professionals work with stroke patients on a daily basis, we expected that they could offer recommendations and comments on SyMPATHys functionalities. Their expectations for and feedback on SyMPATHy were explored through semi-structured interviews.

### B. Participants

In total, 10 participants were involved in this study, including nine health care professionals (six occupational therapists (OTs), two physiotherapists (PTs) and one medical doctor (MD)) and one research engineer (RE), from three rehabilitation centers in the northern part of France: Lille, Le Havre and Evry. The participants included six females and four males aged between 24 and 60 (M = 39, SD = 12) who had worked with stroke patients in the rehabilitation phase since the beginning of their career.

### C. Materials and Procedure

#### 1) Materials

Semi-structured interviews were conducted based on a predefined interview guide (Appendix A). Three main topics were explored with the participants: (i) the concept of a cup for home monitoring, in terms of its acceptability and use in everyday life; (ii) the usefulness of each feature; and (iii) relevant feedback.

#### 2) Procedure

The semi-structured interviews took place in quiet offices within the rehabilitation centers. Following a presentation, participants were informed about the nature and the aims of the interview. The interview was then conducted according to the predefined interview guide. The interview started with a presentation of the SyMPATHy cup, including the concept of continuous monitoring of the arm and hand motor activity of stroke patients at home during everyday life. The functionalities of the cup and the feedback presented to the patient were demonstrated to the participants. Finally, we followed the interview guide presented above. The average duration of each interview was 45 minutes (min: 32 minutes, max: 68 minutes).

### D. Results

A thematic content analysis of the interviews was performed. Based on the topics explored during the interviews, we extracted the following categories:

#### 1) Monitoring of Motor Activity by a Smart Cup

All of the participants mentioned that the use of an ADL to continuously monitor the motor activities of stroke patients within the rehabilitation center and at home was relevant, reporting that health care professionals cannot collect continuous data after the patient leaves the medical center. All participants also appreciated the fact that SyMPATHy can be used in the same way as any other standard cup. However, all participants suggested reducing the size and weight of SyMPATHy in order to facilitate its usage by stroke patients.

#### 2) Liquid Level Indication

Seven participants (four OTs, two PTs, one RE) reported that the liquid level measurements give information that is too complex, since the system reports the quantity of liquid poured, the quantity of liquid the patient drank and the speed of filling. Therapists primarily need to know whether the patient is able to pour water into a cup, that is, the quantity of liquid poured into the cup. However, three OTs felt that the remainder of the information would be useful in assessing the recovery and adapting the rehabilitation program at a later stage, since the filling speed and the quantity of liquid poured into the cup reveal motor skills required for independence in everyday life. As these results were ambiguous, we decided to implement the solution proposed by the majority (i.e. using only the quantity of liquid inside the cup), in order to simplify the liquid level detection functionality. Nine participants (six OTs, two PTs, one MD) mentioned that using visual feedback can be stressful for the patient, as they often cannot control their movements precisely. However, seven participants (five OTs and two PTs) felt that using a discrete system for the liquid level was a clever way to counter visual deficiency. Since these results were also ambiguous, we decided to implement the solution proposed by the majority (i.e. to remove the feedback).

#### 3) Position Detection

All participants reported that the relative position of the cup was very interesting in terms of a positioning exercise with a goal. Health care professionals often ask the patient to move an object (cylinder, cube or prism) to a specific position and assess the accuracy of the task by measuring the distance between the object and the target. Although SyMPATHy assesses the position of the cup, the assessment is binary (i.e. whether or not the target was reached). The majority of the participants (nine in total: five OTs, two PTs, one MD, one RE) would prefer a quantitative assessment of the position of the cup in relation to the target. As for feedback, the majority of the participants (eight in total: four OTs, two PTs, one MD, one RE) judged that the use of binary audio feedback when the cup is placed in the required position was not useful, as patients can see whether the cup is placed on the target. Moreover, the majority of the participants (six in total: three OTs, one PT, one MD, one RE) would prefer no feedback when the target is reached by the patient.

#### 4) Monitoring of the Grasping Force

Measurement of the grasping force was judged to be useful by all therapists. Although monitoring of the force applied on the cup is relevant, the position of the sensor on the hand print was found to be too specific by six participants (four OTs, one MD, one RE). Since patients already have difficulty in grasping an object, grasping the cup with specific finger positions is often impossible. All participants proposed placing sensors all around the cup, in order to detect any hand and finger configuration. Moreover, all participants agreed that a pressure map of the hand would be much more useful in assessing the hand prehension and detecting spasticity. All participants agreed that there should be no feedback for the grasping force, since patients cannot act on it. Spasticity also increases as a result of stress, fever or fatigue, and is therefore likely to alter the grasping force applied on the cup.

#### 5) Orientation of the Cup

Six participants (three OTs, one PT, one MD, one RE) reported that detection of the orientation of the cup does not provide as much information as the authors initially believed. Monitoring only the orientation of the object may be useful, but participants would prefer to detect both motion and velocity in order to assess irregular movements, for example. Nine participants (all except the RE) reported that visual feedback on orientation is likely to introduce stress during manipulation, since patients cannot correct the orientation of the object. They proposed removal of this feedback when monitoring within the rehabilitation center and at home, in order to avoid a potentially stressful experience during the manipulation of the cup.

#### 6) Tremor Detection

Six participants (four OTs, two PTs) noted that assessing the evolution of tremors may lead to the detection of a relapse of motor function, and would mean that therapists could adapt the rehabilitation program in response. Five participants (three OTs, two PTs) reported that the amplitude of the tremor would be more interesting than the frequency, since it would reveal more problems; the tremor frequency is only useful when it is related to the amplitude. Tremor detection therefore seems to be less useful as feedback for the patient. All participants agreed that there is no need for real-time alerts on tremors, since the patient cannot act on these.

### E. Recommendations

The outcomes of this study indicated that the SyMPATHy smart cup had the potential to perform monitoring of the arm and hand motor activities of stroke patients within a rehabilitation center and at home. This preliminary study also showed that the functionalities and feedback provided by the cup needed to be reviewed and adapted to the therapists and patients needs. The participants proposed reducing the size and weight of the cup, and suggested removing both the feedback and the cups position detection functionality, since visual feedback is not useful for the patients and may even frustrate them. In addition to tremor frequencies, the participants proposed displaying the amplitude for each frequency. Moreover, participants reported that the grasping pressure needed to be measurable regardless of the configuration of the hand during grasping. The cup needs to be as transparent as possible in terms of usage, in order to avoid rejection of this technological concept.

## VI. Iteration 2: Improvements and Insight Into the Visualization Application

### A. New Cup Design

**FIGURE 2.**
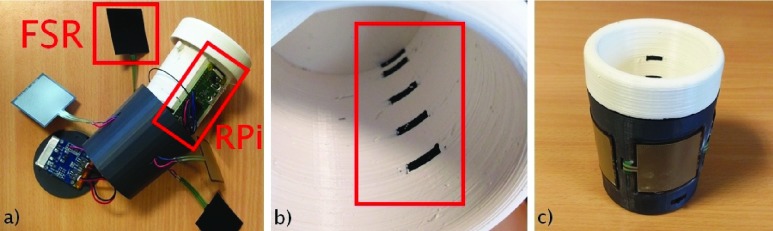
The new cup prototype: a) exploded view with electronics; b) the 3D-printed liquid level sensors; and c) the complete cup.

Based on the recommendations from the preliminary study, a new smart cup was developed. The displays and audio speaker were removed from the new version of SyMPATHy, and new pressure sensors were added around the cup in order to detect grasping in any configuration. Square FSR 406 pressure sensors were placed equidistant from each other in order to cover the whole perimeter of the cup. Then, new liquid level sensors were directly 3D printed into the body of the cup with conductive material, replacing the wires used as electrodes. Thus, the size of the cup was reduced from 16 $\times \,\, 8$ cm to $10\times7$ cm. The weight of the cup was reduced from 338 g to 155 g by removing the electronics from the old functionalities (NFC reader, LEDs etc). The new prototype is shown in Figure 2.

### B. Insight on the Visualization Tool

**FIGURE 3.**
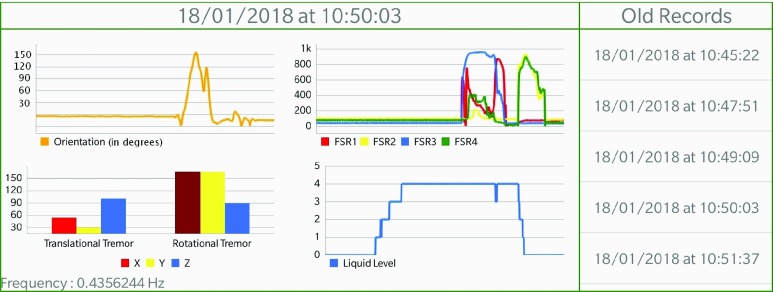
Visualization interface: orientation graph (top left), pressure graph (top right), tremor graph (lower left) and liquid level graph (lower right).

In order to present the data collected by SyMPATHy to the therapists, we designed an accessible visualization tool that allows for easy collection, recording and visualization of data in real time using a tablet. This tool also allows for visualization of previous records on the tablet. We decided to divide the application interface into four sections corresponding to the orientation of the cup, the pressure during grasping, the liquid level and tremors. Line charts were used for the orientation of the cup, the pressure during grasping and the liquid level, since these can present time series information and are the most widely used visualization tool for temporal data. A bar chart was used for translational and rotational tremors, as these are single values computed at the end of the record. Each bar of the tremor graph represents the amplitude of the tremor on the corresponding axis during recording. By clicking on the graph, the user can display the tremor frequency of the corresponding axis. The application interface is illustrated in Figure 3.

After the first visualization tool was designed, a preliminary study was conducted with health care professionals to assess its usability interface and to collect feedback on possible improvements. Three health care professionals who participated in the previous study were involved in this study. The therapists were two females and one male aged between 24 and 35 (M = 27.5, SD = 6.3) who worked with stroke patients in the rehabilitation phase. We presented the tool to these therapists and discussed the interface and the way in which the data were displayed. We also discussed the use and usability of the interface in their medical practice.

The results showed that most of the visualization choices were useful and very well accepted by all the participants. However, two participants reported that the liquid level graph was not the best representation for them. Instead of a line graph, these therapists preferred a pictogram in real time showing that the patient was filling or emptying the cup. They would also prefer to see how long it took to fill the cup, the number of times the cup was filled and emptied, and the period of time for which the cup was held. This information was felt to be more representative of the patients abilities than a line graph corresponding to the real-time liquid level. These modifications will be included in the next version of this application.

## VII. Iteration 3: Preliminary Study With Patients on the Usability and Acceptability of the Cup

### A. Aim of the Study

This study aimed to give preliminary insight into the usability and acceptability of the cup by stroke patients during rehabilitation exercises within a rehabilitation center. We also aimed to carry out a prospective assessment of the usefulness of this device in monitoring motor activity within the rehabilitation center and at home. The study was approved by a national ethical committee “Comité de Protection des Personnes” (ID RCB 2017-A02020-53)

### B. Participants

In total, nine patients were involved in this study: three females and six males aged between 19 and 86 (M = 62.9, SD = 20.6) who had experienced a stroke within the previous 24 months (M = 10.5, SD = 5.7). Six patients had had an ischemic stroke, while three patients had experienced a hemorrhagic stroke. Only one patient was left-handed, and eight patients had impairment in their non-dominant upper limb. The patients who had had an ischemic stroke faced various difficulties directly after the stroke, such as hemiparesis, dysarthria, hemiplegia, ataxia, dysmetria or tremors. The patients who had experienced a hemorrhagic stroke faced difficulties such as minor motor disorders, cognitive problems or hemiparesis. Only one participant who had had a hemorrhagic stroke experienced facial paralysis.

### C. Materials & Procedure

**FIGURE 4.**
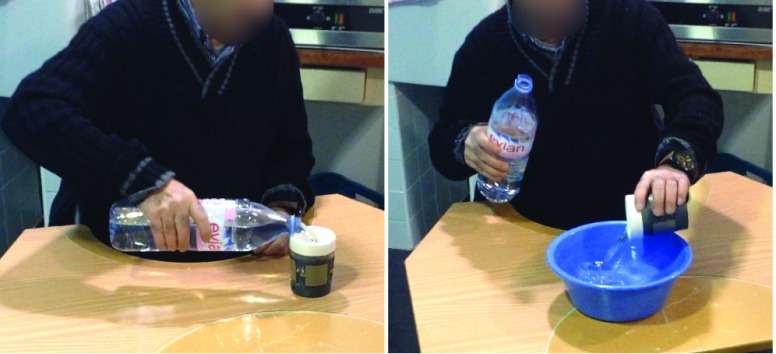
A patient during the experiment.

The experiment was conducted using the second SyMPATHy prototype and took place in a rehabilitation center in Le Havre, France. Each participant was assessed in a quiet room that was separated from other patients. After the patient was given a presentation on the cup, its functionalities and the visualization interface, he/she was informed of the nature and the aims of the study. We asked each patient to sign a consent form and to provide personal information such as the date and the type of stroke. We then presented the experimental protocol. The information requested and the experimental protocol are presented in Appendix B. The protocol consisted of filling SyMPATHy with the healthy arm, grasping it with the paralyzed hand and emptying it into a bowl, using a movement of supination and internal elbow rotation. A bottle of 50 cl capacity and a bowl 10 cm high were used during this experiment (Figure 4). At the end of the experiment, we conducted a semi-structured interview based on a predefined interview guide (Appendix C) to explore the usability and acceptability of the cup to stroke patients.

### D. Results

A thematic content analysis of the interviews was performed. Based on the topics explored during the interviews, we extracted the following categories: (i) acceptability of the cup and applications; and (ii) usability of the cup.

#### 1) Acceptability and Applications

##### a: At the Rehabilitation Center

Eight participants, including six ischemic and two hemorrhagic stroke patients with minor motor impairments, agreed to use the cup during their rehabilitation sessions at the hospital. Most patients reported that even if they could not directly perceive the benefit of such monitoring, the contribution of SyMPATHy for their therapists was useful and could help them to recover faster. However, one participant did not find this device useful for rehabilitation, since this patients recovery was already fairly advanced. This participant mentioned that the device would be more useful during the early stages of post-stroke rehabilitation, when patients still have major motor impairments and are undergoing recovery. Once patients return home, most of them are almost independent and do not need further monitoring. Only two patients proposed exercises which differed from the experiment. A patient who had experienced a hemorrhagic stroke, and who was working on finger movement precision and tactile and sensitive rehabilitation, proposed exercises that involved covering the cup with different textures in order to enhance finger sensitivity. Another patient, who had experienced an ischemic stroke and hemiparesis, suggested to complete the exercise if filling and emptying the cup performed during the experiment with the task of drinking. Indeed, the patient mentioned that the independence for filling and drinking is very important.

##### b: At Home

Six participants agreed that they would use the cup at home during daily life if it helped therapists in terms of monitoring. Five of these had had an ischemic stroke, and the last had had a hemorrhagic stroke; all had experienced hemiparesis. In addition, two participants were willing to use SyMPATHy at home, but thought that the cup should mainly be used in the rehabilitation center during the early stages of recovery. Finally, one older participant (86 y.o.) was not willing to use the cup at home or to make changes to longstanding habits. It is important to note that the most motivated participants in this study were primarily the younger ones (19, 45, 58, 59 y.o.).

#### 2) Usability

##### a: Ease of Use

Eight participants, including six ischemic and two hemorrhagic stroke patients with minor motor impairments, reported that the cup was very easy to use and could be used in the same way as a standard cup. The size and weight were fine, and the placement of the FSR was not a problem during grasping and manipulation. One participant with major upper limb impairment reported that the cup was too heavy and the surface too slippery. Moreover, five participants (four ischemic stroke patients with dysarthria and hemiparesis, and one hemorrhagic stroke patient with minor motor impairments) said that the rim of the cup was too thick and needed to be made thinner.

##### b: Potential Problems

Five participants (three ischemic and two hemorrhagic stroke patients with light motor impairments and hemiparesis) did not experience any potential problems with SyMPATHy and reported that the cup was steady, easy to handle and light. Two participants (ischemic stroke patients with hemiparesis and dysarthria) mentioned that the cup could be spilled during manipulation. However, they reported that this problem also existed with a standard cup, and the spilling of water was not unique to SyMPATHy. Furthermore, three participants (ischemic stroke patients) mentioned the risk of falling and breaking that could also happen with a standard cup. Finally, one participant, an ischemic stroke patient with left upper limb impairments, asked whether the voltage used for the electronics was dangerous.

##### c: Data Transmission

Six participants (five ischemic stroke patients with hemiparesis and dysmetria, and one hemorrhagic stroke patient with hemiparesis) agreed to the transmission of data over the Internet to the rehabilitation center; they did not have concerns about privacy since SyMPATHy data were not judged to be private by these participants. Two participants, including the oldest one (86 y.o.) did not want to transmit their data via the Internet. One of them preferred to record data locally, and brought the USB stick to the rehabilitation center.

### E. Conclusion

This preliminary study provides interesting results from an initial evaluation of the usability and acceptability of the SyMPATHy smart cup. Most of the patients ($\approx 89\%$) involved in this study mentioned that the shape, size and weight of the cup were good and that the cup was easily graspable. The rim was too thick, according to $\approx 56\%$ of the patients. Most of the participants ($\approx 89\%$) reported that the cup would be useful during sessions, and $\approx 67\%$ agreed that the cup would be useful at home during their everyday life. Finally, $\approx 67\%$ of the patients had no concerns about data transmission. The preliminary feedback from the patients in terms of usability and acceptability are promising, and further experiments are required to complete these findings.

## VIII. Conclusion and Perspectives

This paper describes the development of SyMPATHy, a prototype of a self-contained smart cup that can monitor the recovery and independence of stroke patients by providing objective and qualitative information (grasping force, liquid level, orientation and position of the cup) to therapists. The platform design is based on the drinking ADL and allows monitoring of arm and hand motor activities during rehabilitation sessions and at home. Moreover, the therapist can evaluate the appearance and evolution of tremors and adapt the rehabilitation program according to the recovery progress of the patient. Following the implementation of the first prototype, a preliminary study was carried out to collect feedback from health care professionals on possible improvements. The results showed that SyMPATHy was a promising prototype but required several improvements. Based on these results, a new cup prototype was developed, involving the removal of the displays and the detection of the cups position. This resulted in a reduction in the size and weight of the cup. A mobile application for data collection and visualization was also created. Interviews with health care professionals regarding this visualization tool allowed us to collect feedback and improve the visualization interfaces. Finally, a preliminary study was carried out with patients to assess the usability and acceptability of SyMPATHy. The results showed that the majority of patients agreed to use this device during sessions at the rehabilitation center and at home, and the design of the cup did not pose fundamental challenges for the patients except for the rim of the cup, which needs to be made thinner.

Future work will address several issues. New technologies will be investigated to provide a pressure map for the entire surface of the cup, providing more detailed information about grasping pressure and hand and finger positions, as identified in the preliminary study. Furthermore, information on movements such as linear acceleration or translation amplitude could provide complementary data on these movements. Experiments with stroke patients and therapists during rehabilitation sessions and at home that will assess the acceptability and usability of the SyMPATHy cup in more detail are currently planned. We are also planning to investigate the effectiveness of the measurements performed by the cup compared to clinical outcomes that monitor the recovery such as dynamometers or ARAT procotol. Finally, some information cannot be retrieved in the current design, such as the way in which the patient approaches the cup, for example. To overcome this limitation, it would be valuable to combine the cup with a smart garment. The field of smart textiles is growing rapidly, and the creation of a garment with embedded textile sensors to monitor the patients arm and chest configuration could provide relevant information about the period before the cup is grasped. We have already begun work on a smart garment incorporating conductive threads that is able to monitor elbow flexion using machine learning [34].

## Appendix

### A. Preliminary Study Interview

#### 1) Concept Presentation

We first exposed the concept of the cup embedding sensors in order to monitor post-stroke patients activities during ADLs. We presented the cup, its functionalities and its associated sensors. The following questions were posed to the therapists.

• Are ADLs relevant to the continuous monitoring of motor activities at home?
• Do you have any suggestions for the dimensions of the cup prototype?

#### 2) Validation of Functionalities

We then presented each feature in detail, including the type of collected data and the purpose of the feature. The therapists were asked the following questions:

• Is reporting the liquid level as well as the filling speed relevant to the patient assessment? Why?
• Is using the cup to perform positioning exercises relevant to the patient assessment? Why?
• Is reporting the grasping force relevant to the patient assessment? Why?
• What do you think about the sensor placement on the cup?
• Is reporting the orientation of the cup relevant to the patient assessment? Why?
• Is reporting tremors relevant to the patient assessment? Why?
• Would you prefer monitoring of tremor frequencies or amplitudes? Why?

#### 3) Validation of the Feedback

Finally, the associated feedback and the thoughts behind the design choices were presented to the therapists. The following questions were asked:

• Would you prefer discrete or continuous feedback on the liquid level? Why?
• Is the use of binary audio feedback relevant to the relative placement of the cup? Why?
• Would you prefer feedback on the grasping force applied on the cup? Why?
• Is the use of visual feedback relevant to the orientation of the cup? Why?
• Would you prefer feedback on the tremors of the cup? Why?

### B. Experiment Protocol

The patient sits on a chair, and a height-adjustable table is placed in front of the patient, the height of which is adjusted to match the elbow height of the patient when the arms are alongside the body. Once the patient is installed, SyMPATHy and a half-empty opened bottle of capacity 50 cl are placed on the table in front of the patient, spaced at shoulder width. The cup is placed in front of the paralyzed arm, while the bottle is placed in front of the healthy arm. Once the experiment has been set up, the patient is asked to carry out following task sequence:

1 Grasp the bottle with the healthy limb and fill the cup entirely;
2 Put the bottle down on the table;
3 The bottle is replaced with a bowl 10cm high;
4 Grasp the cup and place it above the bowl;
5 Empty the cup into the bowl with a movement of supination and elbow internal rotation;
6 Put the cup down on the table.

Once this sequence is complete, the setup is reset (the bowl is replaced with the half-filled bottle). The patient performs this sequence as many times as possible, according to his/her physical state.

### C. Patient Study Interview

Firstly, we asked for some personal information about the patient, as follows:

• Name
• Surname
• Date of Birth
• Type of stroke (hemorrhagic or ischemic)
• Date of stroke
• Right-handed or left-handed
• Paralyzed limb
• Medical history
• Profession

Secondly, we asked the patient whether he/she would accept this device for rehabilitation, and what kind of applications the patients could imagine for the cup in the rehabilitation center and at home. Patients were asked the following questions:

• Would you like to use this device during your rehabilitation sessions? What kinds of applications can you imagine for SyMPATHy during sessions at the rehabilitation center?
• Would you like to use this device at home?

Following this, usability and acceptability were discussed. Patients were asked the following questions:

• What is your opinion of the ease of use of SyMPATHy (e.g. its size and weight)?
• What potential problems do you see in terms of its use? Do you have any concerns about this object?
• Do you have any concerns about the transmission of collected data?
